# Variation in the response of bovine alveolar lavage cells to diverse species of probiotic bacteria

**DOI:** 10.1186/s13104-020-4921-9

**Published:** 2020-03-17

**Authors:** Susan D. Eicher, Carol G. Chitko-McKown, Keith A. Bryan

**Affiliations:** 1 USDA-Agricultural Research Service Livestock Behavior Research Unit, West Lafayette, IN 47907 USA; 2 USDA-Agricultural Research Service U.S. Meat Animal Research Center, Clay Center, NE 68933 USA; 3 Chr. Hansen, Inc., Animal Health & Nutrition, Milwaukee, WI 53214 USA

**Keywords:** Probiotic, Lung, Bovine, Phagocytosis, Leukocyte

## Abstract

**Objective:**

Probiotics are fed to improve enteric health, and they may also affect respiratory immunity through their exposure to the upper respiratory tract upon ingestion. However, their effect on the respiratory system is not known. Our aim was to determine how probiotics affect functions and markers of bronchoalveolar lung lavage cells (BAL) isolated from lungs of calves at slaughter.

**Results:**

Treatments consisted of ten probiotic species and one control treatment. Probiotics and BAL were incubated 1:1 for 2 h at 37 °C and 5% CO_2_. The cell surface markers measured included CD14, CD205, and CD18, and *E. coli* bioparticles were used to measure phagocytosis and oxidative burst. Differences were considered significant at *P *≤ 0.05 and were noted for percent cells fluorescing and mean fluorescence intensity for CD14 and CD205. Additionally, oxidative burst was different as measured by both percentage of cells fluorescing and mean fluorescence intensity, and phagocytosis differed among species as measured by mean fluorescence intensity. Overall, probiotic species differed in their ability to suppress or increase leukocyte function showing that probiotic bacteria differentially modulate BAL.

## Introduction

Probiotics have been investigated for many health benefits, however little research has investigated their effects on the respiratory immune cells [1]. Lungs were previously believed to be a sterile environment, but that has recently been revealed to not be the case in the upper respiratory tract [2]. Others have shown a communication between the gut and respiratory immune mechanisms [3–5]. Because most applications of probiotics are by an oral route, we hypothesized that some of the probiotic may be having an impact on the respiratory system as well as the intended enteric system [5, 6]. As an initial step, our aim was to determine how bronchial alveolar lavage cells (BAL) responded to eight potential probiotic microbes and two synbiotics designed for livestock.

## Main text

### Methods

Lungs were recovered from 5 beef calves after slaughter and placed on ice for transport to the laboratory approximately 10 min away. The lungs were lavaged with 100 mL of warm (37 °C) HBSS (Gibco, ThermoFischer). A minimum of 50 mL of lavage fluid was obtained with 2 washes. The lavage fluid was filtered over sterile gauze into a 50 mL sterile tube. The tubes were centrifuged at 1800×*g* for 15 min at 4 °C. Supernatants were discarded, and BAL were resuspended in 10 mL of cold sterile HBSS, then centrifuged a second time. Supernatants were discarded, and cells were resuspended in RPMI + glutamine at 10^6^ cells/mL. The synbiotics (probiotics plus prebiotics) used in this study for BAL stimulation were US (a 3-strain *Lactobacillus* probiotic, USDA-ARS), and Probios^®^ symbiotic (PB) (Vets Plus, Inc., Menomonie, WI). Eight other single microbial probiotics were obtained from Chr. Hansen, Inc., including *Lactobacillus animalis* LA-51, *Propionibacterium freudenreichii* PF-24, *Enterococcus faecium* CH-212, *E. faecium* SF-273, *E. faecium* M-74, *Bifidobacterium animalis* ssp. lactis BB-12, *Bacillus subtilis* EB-15, and *B. amyloliquifaciens* ZM-16. Lavage cells and bacterial cells were incubated 1:1 for 2 h at 37 °C and 5% CO_2_ in RPMI + Glutamine. One 15 mL polyproylene tube contained 1 mL of the 1 ×10^6^ cells/mL of BAL and 1 mL probiotic or synbiotic to deliver a 1:1 MOI, and one tube was left as cells only control (CNT) with 1 mL media. Cells (500 µL) were then aliquoted into flow cytometry tubes (12 × 75 mm polypropylene, Falcon, Corning, NY). Three μl of the following antibodies were added to each of the tubes; medium (cells only); anti-bovine CD14 (fluorescein isothiocyanate (FITC), BioRad, Hercules, CA) and anti-bovine CD205 (R-phycoerythrin (RPE), BioRad); anti-bovine CD18 (Bov 2030, Washington State University, labeled with Alexa Flour 647), and opsonized *E. coli* bioparticles (Life Tech. Corp E2870 and P35361). After a 1 h incubation at 37 °C in a shaking water bath, cells were washed twice with the addition of 1 mL of 1× HBSS, centrifuged at 3000×*g* for 3 min. Fluorescence was determined using the BD Fortessa (Beckman Coulter, Brea, CA) with excitation set at 488 nm and 640 nm and emission evaluated at 530 nm (FITC), 575 nm (R-PE), and 647 nm (Alexa Fluor 647). Data were collected for the total BAL population. To determine differences among the probiotic/symbiotic treatments, statistical analyses were performed using Mixed models in SAS (version 9.4; Cary, NC) with probiotic treatment as the fixed effect. Mean separations were by Bonferroni testing.

### Results

**Fig. 1:**
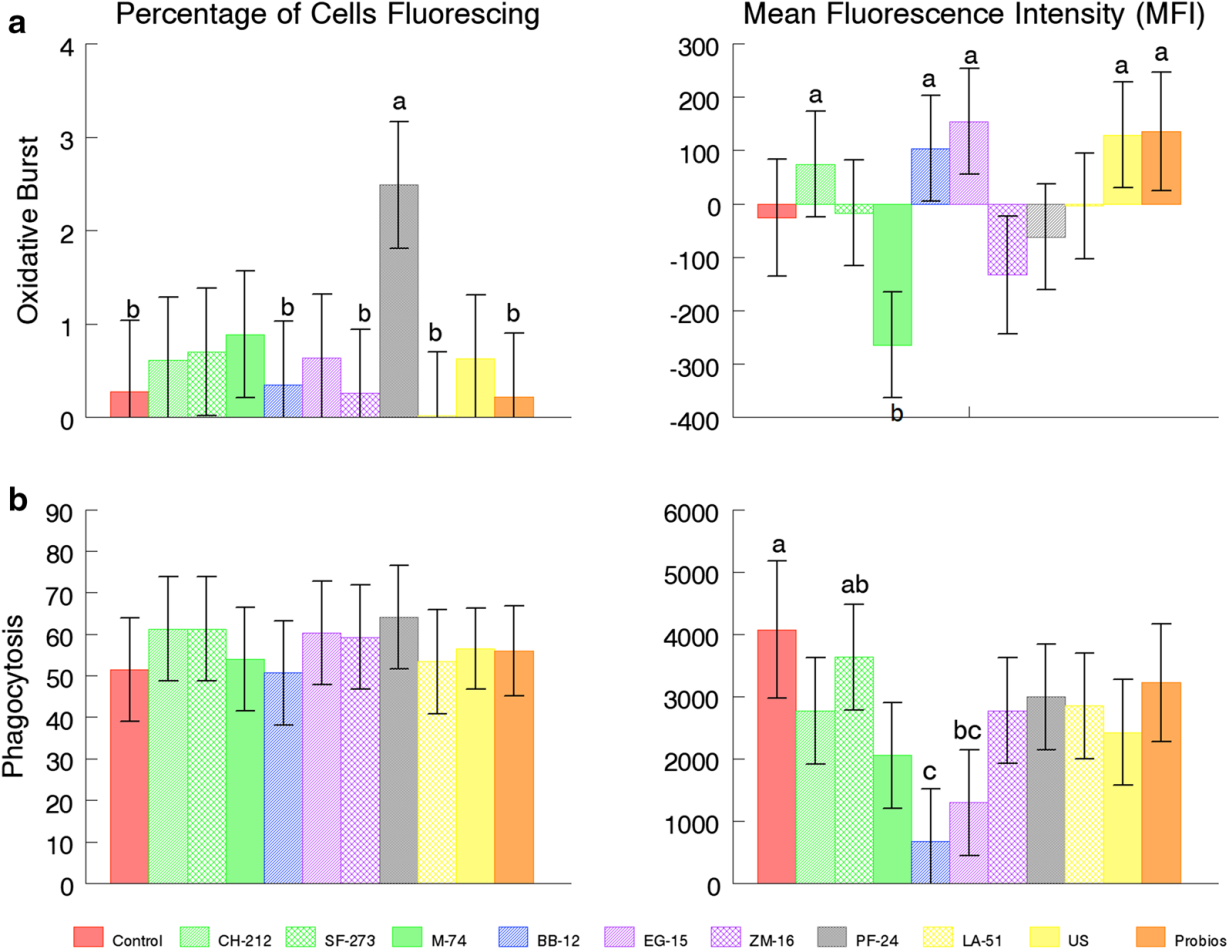
Oxidative burst (**a**) and phagocytosis (**b**) by total lung lavage cells (BAL). ^a, b, c^ designate differences between means of the probiotic stimulants (*P *≤ 0.05)

**Fig. 2:**
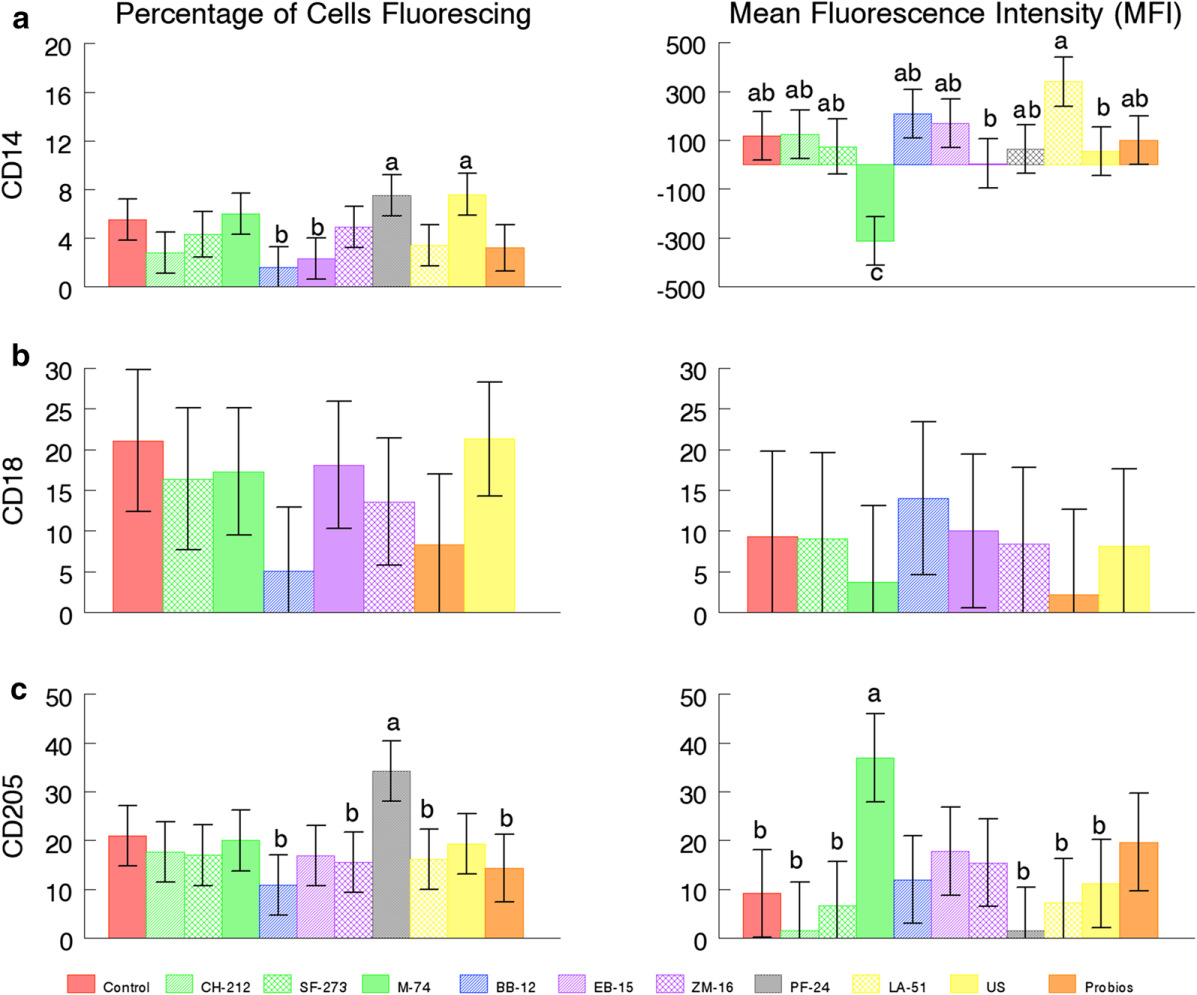
CD14 (**a**), CD18 (**b**), and CD205 (**c**) expression of total lung lavage cells (BAL). The left panel of each cell marker is the percentage of cells expressing that marker and the right panel is the mean fluorescence for each marker. ^a, b, c^ designate differences between means of the probiotic stimulants (*P *≤ 0.05)

Cell counts determined that BAL were > 60% macrophages. Preliminary data were used to determine that a 1:1 ratio of the probiotic to leukocyte was appropriate for a good response. Differences were considered significant at *P *≤ 0.05. Mean fluorescence of phagocytosis of *E. coli* bioparticles was less for *B. animalis* BB-12 and *B. subtilis* EB-15 than CNT, but percentage (%) of cells phagocytizing was not significantly different (Fig. 1a). Mean fluorescence of oxidative burst by *E. faecium* M-74 was less than *E. faecium* CH-212, *B. animalis* BB-12, *B. subtilis* EB-15, PB, and US, but none were significantly different than CNT. The % of cells with oxidative burst was greater for *P. freudenreichii* PF-24 than for *L. animalis* LA-51, *B. animalis* BB-12, *B. amyloliquifaciens* ZM-16, PB, and CNT (Fig. 1b). CD14 mean fluorescence was least for *E. faecium* M-74 compared with all other microbes and CNT, but % of cells expressing CD14 was greatest on *P. freudenreichii* PF-24 and US compared with *B. subtilis* EB-15 and *B. animalis* BB-12, but not CNT. CD205 mean fluorescence was greatest for *E. faecium* M-74 compared with *L. animalis* LA-51, *P. freudenreichii* PF-24, *E. faecium* CH-212, *E. faecium* SF-273, CNT, and US. Percentage of cells expressing CD205 and CD18 was not significantly different from CNT (Fig. 2).

### Discussion

Although there is increasing literature on probiotics and the intestinal microbiome, there are few studies on the effects of probiotics on respiratory immunity in cattle. Lima et al. [7] showed the changes in healthy and diseased calves’ upper respiratory tract microbiome from 3 days of age to 35 days of age. Bosch et al. [8] suggested that imbalances of the upper respiratory tract microbiome may lead to invasion by and overgrowth by pathogenic bacteria. Homan et al. [1] determined that the microbiome of cattle on the day of arrival into a feedlot and after 60 days were significantly different. Corbett et al. [9] noted that feeding probiotics did not reduce respiratory susceptibility in cattle. However, Adjei-Fremeh et al. [6] reported that feeding of probiotics induced global gene expression upregulation of genes associated with both innate and adaptive immunity. Cytokines, chemokines, TLRs, and stress-related signaling molecules that are related to the inflammatory response and to the maintenance of homeostasis were predominant. The cattle used in this study had undergone transport stress and movement into the abattoir prior to our samples, thus this could have resulted in cortisol release altering immune profiles and functions.

In this in vitro work we sought to determine whether probiotic microbes could stimulate the immune functions of leukocytes obtained by lung lavage by changes in cell surface markers. We measured CD14 as part of the LPS recognition molecule, CD18 as a marker of cell activation and adhesion, CD205 to determine the role of dendritic cells, and phagocytosis of *E. coli* bioparticles and the associated oxidative burst to determine phagocytic function. Most literature reports on *Lactobacillus* strains in disease prevention of pneumococcal infections [3] and *Lactobacillus* have been used to determine some of the mechanisms that reduce susceptibility in vitro [10]. However, *Bacillus subtilis* delivered intranasally increased TLR expression in tonsils of pigs [11]. Monocyte derived DCs were not affected in numbers or maturation by the soluble mediators of *Lactobacillus rhamnosus*, but their capacity to modulate T cell responses was enhanced [12]. Additionally, *L. rhamnosus* CLR 1505 modulated the TLR3-mediated immune response in the respiratory tract of mice [13]. Lehtoranta et al. [14] reviewed some common probiotics’ effectiveness in humans and mice. They concluded the variability in outcomes may be attributed to the strains of probiotic in use, bacterial dose provided, and additives contained within the probiotic products. TLR3 is an important component in the inflammatory response to viral infection, and with the associated pathology. Our data showed an increase in the number of cells expressing the CD205 dendritic cell marker for *P. freudenreichii* PF-24 compared to other probiotic microbes, but it was not statistically different than the control cells. *Lactobacillus animalis* LA-51, *B. animalis* BB-12 and *B. amyloliquifaciens* ZM-16, and the Probios product showed a decrease in the number of CD205-expressing cells compared to *P. freudenreichii* PF-24. In contrast, the mean fluorescence of CD205 was greatest for *E. faecium* M-74 compared to CNT, but *Lactobacillus* such as LA-51 and US (3 strains of *Lactobacillus*) both resulted in lower CD205 mean fluorescence than *E. faecium* M-74 and similar to CNT. In concurrence with Forsythe’s [15] observation that microbes have effects on dendritic cell phenotype and function, our data show that dendritic cells are certainly playing a role in the ability of the leukocytes to modulate immunity. The increase in the % of cells with oxidative burst corresponds with the increase in the % of cells expressing the DC marker. This would be a desirable characteristic of a probiotic affecting the respiratory tract.

The recognition of gram negative bacteria requires the expression of CD14 as part of the LPS recognition molecule which it binds only in the presense of LPS-binding protein. In the current in vitro study, differences among the probiotic microbes were evident in the % of cells expressing CD14 molecules (no differences from CNT), but *E. faecium* M-74 CD14 fluorescence was reduced compared to all other treatments and this corresponds to the decrease in oxidative burst due to *E. faecium* M-74 microbe stimulation.

Nasally delivered *L. lactis* NZ900 improved clearance of *S. pneumoniae*, possibly by a competitive exclusion mechanism [3] and by enhanced IgA and IgG in BAL fluid in mice. Marranzion et al. [16] demonstrated TNF-α concentration was not altered in BAL compared with serum and intestinal fluid, but IFN-γ was increased by 2 or 3 strains of *Lactobacillus* compared to controls in BAL, both in ex vivo and in vitro experiments.

The oxidative burst of those 2 strains was also greater than controls [16]. Cell counts of pathogenic *C. albicans* in lungs of infected mice showed a reduction with *L. casei* CRL431 and *L. rhamnosus* CRL1505 treatments. In contrast, our data show only suppressed fluorescence of phagocytic activity by *B. animalis* BB-12 and *B. subtillus* EB-15 compared to controls, and no differences were evident in the number of cells that were phagocytizing. These microbes also decreased the % of CD14 expressing cells, demonstrating the importance of the CD14 molecule in phagocytosis of the *E. coli* bioparticles. We did see enhanced number of cells with oxidative burst by *P. freudenreichii* PF-24 compared to controls and to 4 other probiotic microbes. Oxidative burst fluorescence was not different from controls for any treatment, but differences among the treatments that had enhanced fluorescence (*E. faecium* CH-212) and with suppressed fluorescence (*E. faecium* M-74) were evident. There are numerous differences in the approaches used in these two studies including different species, method of probiotic delivery and duration of treatment. Marranzino et al. [16] did much of their study in vivo in mice. Our work in contrast used harvested bovine BAL and tested their responses ex vivo. CD14 changes in *P. freudenreichii* PF-24 were also reflected by enhanced number of cells with oxidative burst, and similarly the suppression of CD14 fluorescence by *E. faecium* M-74 was reflected in reduced oxidative burst. It appears that there are many facets of the BAL interaction with various probiotic microbes that show the variation in whether their interaction will be favorable. *Bifidobacterium animalis* BB-12 benefits for upper respiratory infections in humans were dependent on timing [17]. Method of delivery and duration of supplementation have been cited as reasons for difference in the effectiveness of probiotic supplements on upper respiratory symptoms, some showed benefit in rate while others showed a reduction in duration or severity but not on incidence.

Because we used a static system, in vitro, we would not expect large shifts in cell population percentages such as in our phagocytosis data where little change was evident in the % of cells, but the mean expression showed some substantial differences. It is possible that effects in vivo may be more dramatic because of the increased chance to affect the cell population development.

Other benefits attributed to probiotics are increased expression of mucin genes and mucin secretion in intestines [18], and antimicrobial peptide producing cells, whether that is true for respiratory mucosal surfaces is not known. Additionally, many probiotics have mechanical actions that are antagonistic to pathogens [19].

### Conclusion

*Propionibacterium freudenreichii* PF-24 and *E. faecium* M-74 were most immunomodulatory probiotic microbes compared to CNT. *Enterococcus faecium* M-74 appeared to suppress, and *P. freudenreichii* PF-24 to increase leukocyte functions, showing that probiotic bacteria differentially modulate BAL. Our results indicate that probiotic microbes vary in their immunomodulatory effects, therefor selection of an appropriate probiotic microbe is critical to obtaining the desired immunologic response and outcome.

### Limitations

- N of 5.
- Performed ex vivo.
- Abbreviations
  - BAL: bronchoalveolar lavage cells
  - US: 3-strain *Lactobacillus* probiotic
  - PB: Probios^®^ symbiotic
  - CNT: control
  - FITC: fluorescein isothiocyanate FITC
  - RPE: R-phycoerythrin
  - %: percentage

## Data Availability

The datasets used and/or analyzed during the current study are available from the corresponding author on reasonable request.
